# Continuous Consecutive Reactions with Inter‐Reaction Solvent Exchange by Membrane Separation

**DOI:** 10.1002/anie.201607795

**Published:** 2016-09-27

**Authors:** Ludmila Peeva, Joao Da Silva Burgal, Zsofia Heckenast, Florine Brazy, Florian Cazenave, Andrew Livingston

**Affiliations:** ^1^ Department of Chemical EngineeringImperial College LondonExhibition RoadLondonSW7 2AZUK; ^2^ Department of ChemistryImperial College LondonExhibition RoadLondonSW7 2AZUK

**Keywords:** flow chemistry, homogeneous catalysis, membranes, solvent exchange, synthesis design

## Abstract

Pharmaceutical production typically involves multiple reaction steps with separations between successive reactions. Two processes which complicate the transition from batch to continuous operation in multistep synthesis are solvent exchange (especially high‐boiling‐ to low‐boiling‐point solvent), and catalyst separation. Demonstrated here is membrane separation as an enabling platform for undertaking these processes during continuous operation. Two consecutive reactions are performed in different solvents, with catalyst separation and inter‐reaction solvent exchange achieved by continuous flow membrane units. A Heck coupling reaction is performed in *N*,*N*‐dimethylformamide (DMF) in a continuous membrane reactor which retains the catalyst. The Heck reaction product undergoes solvent exchange in a counter‐current membrane system where DMF is continuously replaced by ethanol. After exchange the product dissolved in ethanol passes through a column packed with an iron catalyst, and undergoes reduction (>99 % yield).

The production of typical active pharmaceutical ingredients (APIs) involves multiple reaction steps with separations (workup) between successive reactions, and is dominated by batch operations. However pharmaceutical manufacturers are actively investigating converting their processes into continuous production, thus seeking cost savings of 10 to 20 % as compared to batch manufacturing,^1^ reduced energy and carbon footprints, and improved overall safety.^2^ In contrast to batch processing, multistep reaction sequences can be conducted employing several flow reactors in series, combined with packed‐bed materials chemically functionalized with catalysts, or reagents for exploiting purification with solid‐phase scavengers, chromatographic separation, or liquid/liquid extraction. A benefit is that intermediates are not isolated but are directly transferred into the next flow reactor.^3^

The optimization of a multistep flow process is challenging. Each reactor unit has to be designed to ensure compatibility with the subsequent unit in terms of flow rate, temperature, and solvent environment. In reality, synthetic sequences are usually split into two or more shorter sequences with product isolation occurring between the sequences. One reason for dissecting a multistep flow synthesis can be the need for a switch between solvents.3a Solvent exchange by distillation is straightforward when the solvent to be removed has a lower boiling point than the replacement solvent, and semi‐batch^4^ and continuous^5^ solvent exchange from low‐ to high‐boiling solvent in flow has been demonstrated. However, a solvent exchange in the opposite direction (reverse boiling‐point order) is typically difficult, and is associated with significant energy consumption and large quantities of intermediate solvent mixtures. Aside from economic effects, thermal operations may degrade the APIs and/or catalysts if they are thermally labile. Reverse boiling‐point‐order solvent exchange has been reported by catch‐and‐release techniques in which the desired product of a solution‐phase reaction is selectively trapped onto a functionalized support material. The compound is subsequently released from the support by pumping in the replacing solvent along with an appropriate releasing agent.3a, 6a However, this approach relies on batch trap and release cycles, thus introducing operating and control complexity.

Furthermore, if two reaction steps utilize different catalysts which can interfere with each other, catalyst removal is essential between sequential stages. Catalyst incompatibility can be minimized by using solid‐phase‐bound and/or immobilized catalysts.^6^ However there are limitations on the practicality of these systems.^7^ Thus in many cases the use of homogeneous catalysts is favored together with an appropriate catalyst recycling technique,^8^ such as using scavenging columns or scavenging agents in solution, liquid–liquid biphasic conditions, or organic solvent nanofiltration (OSN).

Membrane unit operations are well suited for continuous processes because of their ease of operation in flow, scalability, and the absence of phase transitions or biphasic systems. OSN has been demonstrated for solvent exchange^9^ and catalyst recovery for individual processes utilizing predominantly model compounds. Herein we present the first example of continuous consecutive reactions where the catalyst recovery and the solvent exchange are achieved in membrane units. As a case study we have selected two consecutive reaction steps from the synthesis of the API [6‐chloro‐2‐(4‐chlorobenzoyl)‐1H‐indol‐3‐yl]‐acetic acid, a selective cyclooxygenase 2 (COX‐2) inhibitor, steps which require a reverse boiling‐point‐order solvent exchange from DMF to ethanol^10^ (Figure 1). The synthesis reported in the literature has been performed in batch, and the inter‐reaction protocol used for purification and solvent exchange is tedious. Figure 1 shows it comprises seven steps involving a variety of solvents. We replace this process with a single step performed in a membrane cascade unit, and the details are presented in Figure 2.

**Figure 1:**
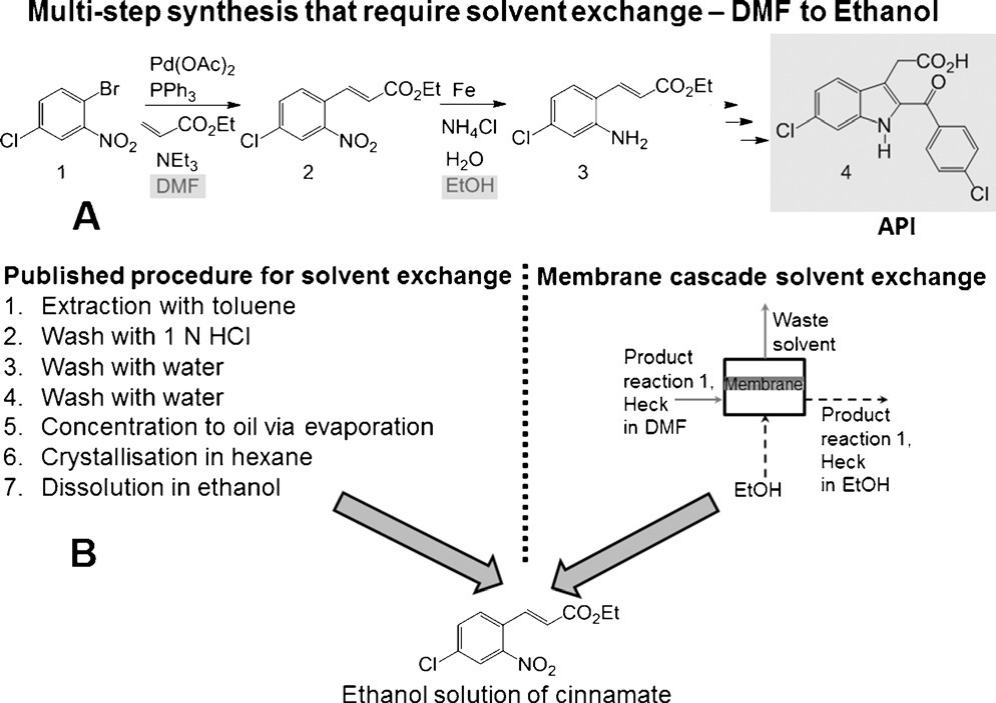
A) Reaction scheme for API synthesis. B) Schematic representation of the alternative routes for solvent exchange. DMF=*N*,*N*‐dimethylformamide.

**Figure 2:**
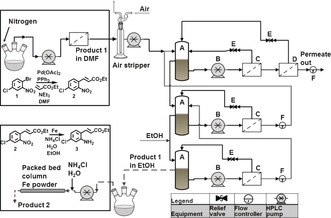
New concept for a continuous process where the Heck reaction and the solvent exchange are performed in continuous membrane units.

The first reaction, a palladium‐catalyzed Heck coupling, was performed in a continuous unit consisting of a plug flow reactor and completely stirred membrane reactor/separator in series^11^ (PFR‐*m*‐CSTR; Figure 3). Although the conversion in the PFR reactor is relatively low, our previous studies have shown that successful pre‐activation of the catalyst occurs within the PFR.^11^ Preliminary kinetic experiments were performed to determine the effect of catalyst loading on the reaction rate and estimate the minimum residence time (RT) achievable in the reactor. As can be seen from Figure S1 (see the Supporting Information), increasing the catalyst loading above 10 mol % does not further accelerate the reaction rate (apparent kinetic constant max 0.66 h^−1^), so our study focused on catalyst loadings within the range of 0.05 to 10 mol %. A simplified kinetic model was developed in order to predict the reactor performance (Figure S1). As can be seen from Figure 3, the shortest RT achievable in the reactor at about 95 % conversion is 10 hours. Reducing the RT to 5 hours resulted in a sharp drop in conversion, as predicted by the model. It was also possible to achieve conversion above 95 % at lower catalyst loadings, however at the expense of a long RT of 53 hours (see the Supporting Information). The palladium concentration in the permeate remained within the expected range (<10 % of concentration in the membrane reactor and below 100 ppm). The palladium contamination of the product is high (ca. 1880 mg Pd kg^−1^ product, at 10 mol % catalyst loading) and the final product will need additional purification. However this product contamination is still about two times lower than in the process performed in batch (ca. 4400 mg Pd kg^−1^ product).

**Figure 3:**
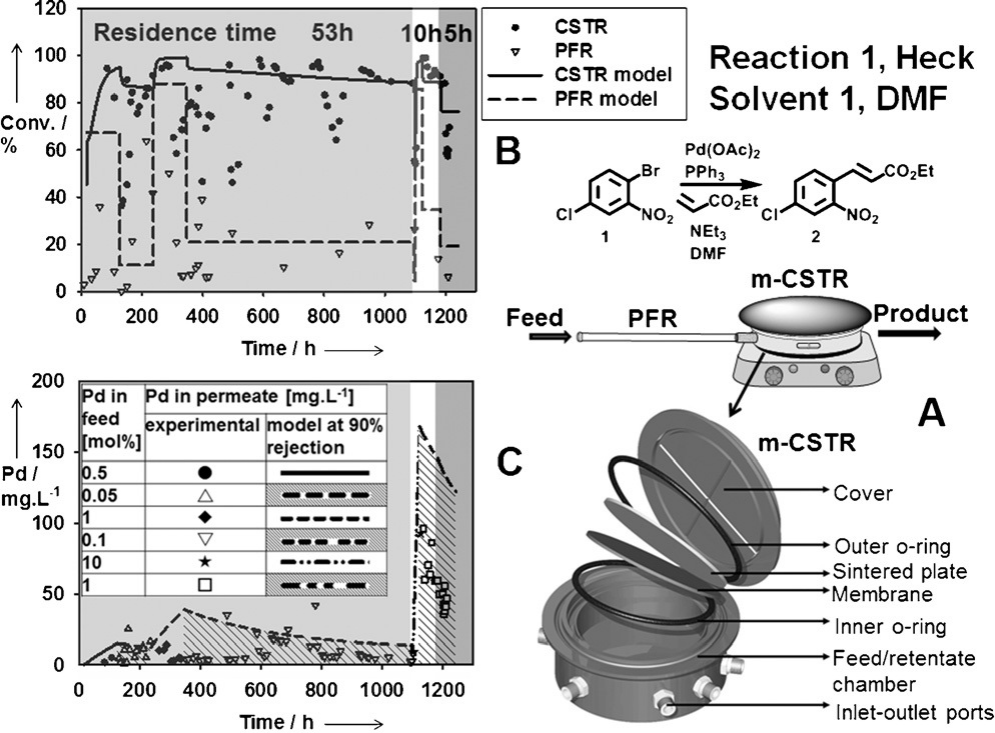
A) Schematic representation of the PFR‐*m*‐CSTR configuration. Conversion in the PFR and CSTR (B) and palladium concentration in the permeate stream from the *m*‐CSTR (C) over time at different residence times and catalyst loadings.

Despite the high catalyst loading used, the product stream was clear and transparent since the bulk of the palladium was retained in the membrane reactor (see Figure S2). The product stream from the *m*‐CSTR was passed through an air stripper to remove residual TEA and ethyl acrylate. The product stream obtained at 53 hours RT was further diluted with DMF to bring the flow rate up to 0.006 L h^−1^ (0.003 L h^−1^ was too low for reliable cascade operation), and passed through the membrane cascade for solvent exchange. This initial experiment was performed to evaluate the cascade operational parameters and also the membrane stability. The cascade was operated in a counter‐current mode9c with the postreaction mixture being fed in at Stage 1 and the replacement solvent fed in at Stage 3 (feed‐to‐replacing solvent ratio 1:2.5; Figure 2). The final product dissolved in the replacement solvent was collected as an overflow from Stage 3. The cascade was operated for about 160 hours with consistent membrane flux and rejection. The solvent was exchanged from 100 % DMF to 82 % EtOH with product dilution of 2.1 and product yield greater than 95 % (Figure 4; see Figure S7A).

**Figure 4:**
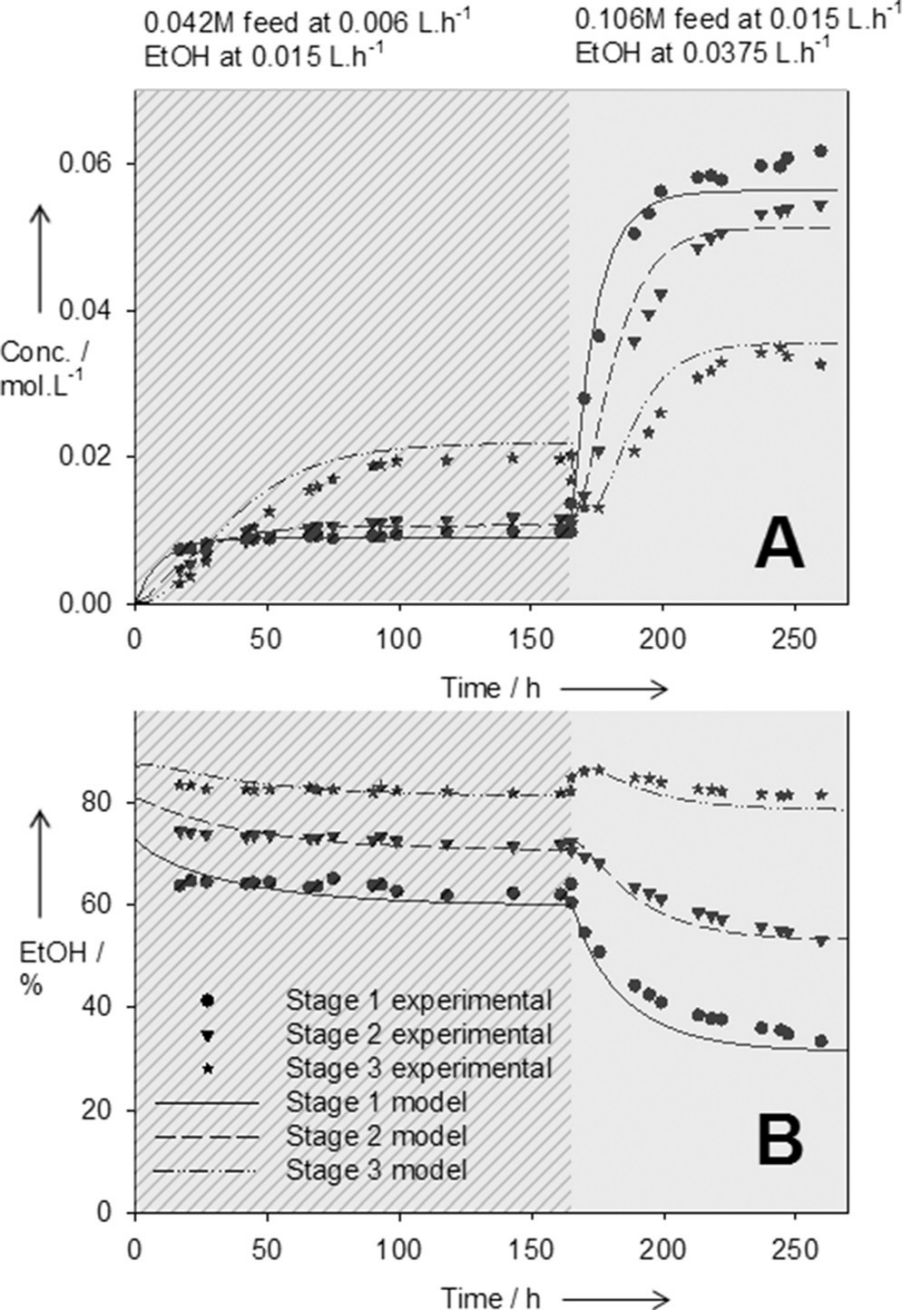
Theoretical and experimental concentration of product (A) and ethanol (B) as a function of time in the membrane cascade stages.

Finally we evaluated the effect of residual DMF, present after solvent exchange, on the second reaction. A series of batch kinetic experiments were performed varying the DMF concentration in the reaction mixture. Results indicated that DMF concentrations of up to 30 % do not significantly affect reaction rate (Figure 5 B), thus the 18 % residual DMF achieved by solvent exchange was sufficient. To further prove the concept, a packed‐bed column with iron was operated in continuous mode, initially using a model solution of 0.05 m **2** in 82 % EtOH and 18 % DMF. The flow rate through the column was adjusted to match the product flow leaving the solvent exchange stage (RT≈52 min). Initially the conversion was incomplete with large fluctuations in the conversion numbers, and the RT in the column was increased to 110 minutes. After about 20 hours of operation the conversion increased to approximately 100 % and remained stable. The model solution was replaced with the cascade product stream initially at 110 minutes RT, and since the conversion remained stable the RT was decreased to 50 minutes. Again the conversion remained steady at about 100 % (Figure 5 C). This outcome confirmed the robustness of the second reaction and that the extent of DMF removal in the solvent exchange was sufficient. The initial fluctuations were attributed to a pre‐activation period^12^ of the iron in the column, and possibly some initial non‐uniformity of the packed bed.

**Figure 5:**
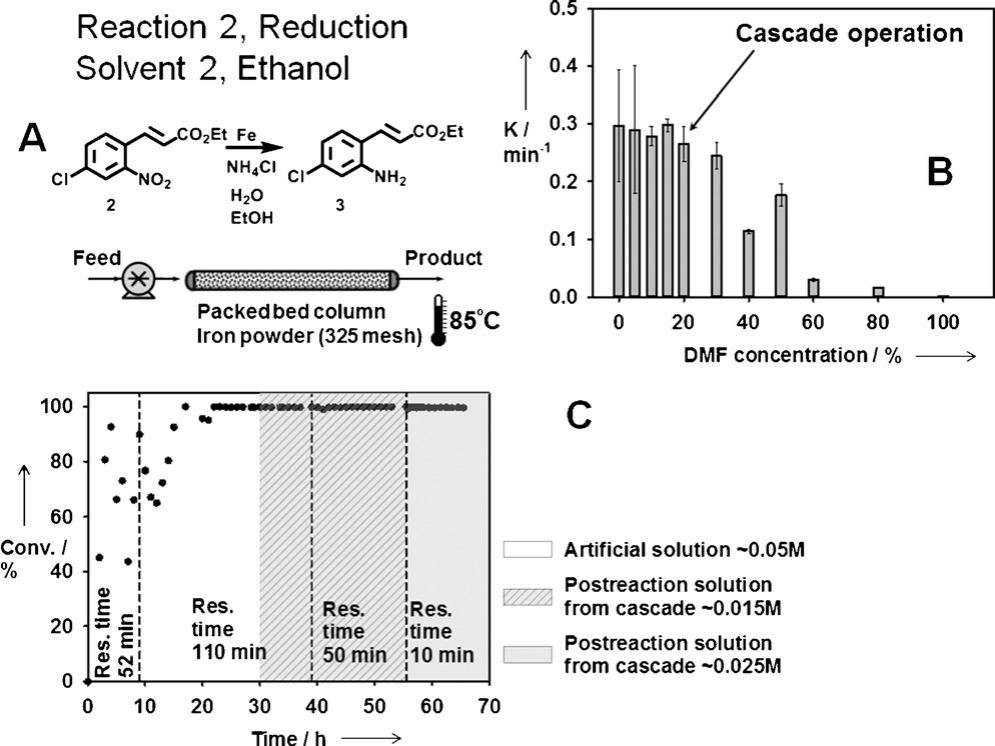
A) Schematic representation of the packed‐bed column. B) Reduction reaction kinetic constant as a function of DMF concentration. C) Conversion in the packed‐bed column overtime at different RTs.

The final challenge was to push the system to its limits by minimizing the RT. Maximum product flow rate and therefore the RT, is determined by the Heck reaction, and as mentioned earlier, the minimum RT in the PFR‐*m*‐CSTR required for greater than 95 % conversion is 10 hours (flow rate 0.015 L h^−1^). After passing through the gas stripper the Heck product stream was fed into the membrane cascade, now without any dilution. A counter‐current stream of EtOH was fed in at Stage 3 of the cascade at 0.0375 L h^−1^ (1:2.5 ratio). Because of the higher concentrations of the retained species, the fluxes through the membrane were lower than that of the first run, thus leading to a slightly higher dilution of the product stream (3 times vs. 2.1 at the first run). The solvent exchange was successful, thus transforming the stream from 100 % DMF to 82 % EtOH with a product yield of greater than 99 %. The cascade product stream was mixed with 0.59 m NH_4_Cl aqueous solution at a ratio of 3.5:1 and passed through the packed‐bed iron column. Despite the short 10 minutes RT in the column the reaction proceeded to about 100 % completion. The products of the first and the second reaction were verified by ^1^H NMR spectroscopy. The purity of the crude reaction mixture was greater than 80 %. Unfortunately the membrane selectivity of *m*‐CSTR was not sufficient to entirely separate the product of the Heck reaction from the side product Et_3_NBr, unreacted substrate (ca. 5 %), and the small amount of the catalyst debris. The impurities were carried through the solvent exchange and fed into the second reaction without any significant impact. In the event of more sensitive reactions, an additional continuous membrane purification unit could be provided (see Figure S10). This study proves that a continuously operated OSN membrane cascade can successfully achieve inter‐reaction reverse boiling‐point‐order solvent exchange. Process and membrane optimization could further improve product quality and flexibility of the membrane cascade (see Figures S8 and S9).

In summary we have demonstrated a continuous process that integrates chemical synthesis with catalyst recovery and reverse boiling‐point‐order solvent exchange by using OSN technology. Significantly, the solvent exchange, usually a cumbersome process which typically requires energy intensive and/or biphasic or semi‐batch operations, was performed smoothly in continuous mode and the product was successfully processed in the next reaction step. The membrane unit operations showed excellent operational stability over a prolonged period of more than two months.

## Supporting information

As a service to our authors and readers, this journal provides supporting information supplied by the authors. Such materials are peer reviewed and may be re‐organized for online delivery, but are not copy‐edited or typeset. Technical support issues arising from supporting information (other than missing files) should be addressed to the authors.

Supplementary
