# Supplementary material for: Continuous Consecutive Reactions with Inter‐Reaction Solvent Exchange by Membrane Separation
Source: Angew Chem Int Ed Engl. 2016 Sep 27;55(43):13576–9. doi: 10.1002/anie.201607795 (PMC5113664; doi:10.1002/anie.201607795)
Supplement: Supplementary file 1 — Supplementary [file ANIE-55-13576-s001.pdf]

Angewandte Chemie
Eine Zeitschrift der Gesellschaft Deutscher Chemiker

Supporting Information

Continuous Consecutive Reactions with Inter-Reaction Solvent Exchange by Membrane Separation

*Ludmila Peeva, Joao Da Silva Burgal, Zsofia Heckenast, Florine Brazy, Florian Cazenave, and Andrew Livingston**

anie_201607795_sm_miscellaneous_information.pdf

Supporting information

Experimental Section

Materials

Analytical grade palladium (II) acetate [$Pd(OAc)_2$], triphenylphosphine (PPh_3), 1-Bromo-4-chloro-2-nitrobenzene (1), ethyl acrylate, anhydrous *N*,*N*-dimethylformamide (DMF), triethylamine (NEt_3), ethyl acetate, diethyl ether, nitric acid (69 %) hydrochloric acid (37 %) and iron powder (325 mesh) were all purchased from Sigma-Aldrich and used as received.

The PEEK membrane used was obtained by dissolving VESTAKEEP® 4000P at a concentration of 12 wt. % and dried from water at 120 °C following the procedure described by Burgal *et al* [1]

Heck Reaction - Product 1 – Ethyl (*E*)-3-(4-chloro-2-nitrophenyl)acrylate (2)[2]

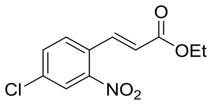

Kinetic study on the effect of catalyst loading

To a solution of 1-Bromo-4-chloro-2-nitrobenzene (1) (0.336 g, 1.42 mmol), $Pd(OAc)_2$ (0.05-10 mol%) and PPh_3 (0.1-20 mol%) in dry DMF (4 mL) in a two-necked flask equipped with a water-condenser was added Et_3N (0.67 mL, 1.98 mmol) and ethyl acrylate (1.52 mL, 14.2 mmol). The solution was heated at 90 °C for 4-48 h. Samples (0.1 ml) were taken periodically and diluted with 0.5 ml DMF, before analysis by GC. For simplicity the reaction kinetic was approximated by a first order reaction in the limiting substrate 1-Bromo-4-chloro-2-nitrobenzene (1) and the kinetic

constant for each catalyst loading was determined using the isolation method. Experiments at each catalyst loading were performed in duplicate and the average value of the kinetic constant was used. An exponential curve fitting was applied in order to describe kinetic constant as a function of the catalyst concentration. This equation was further used in a mathematical model to describe the PFR-m-CSTR performance. Conversion over time at different catalyst loadings and the fitted curve for kinetic constant as a function of catalyst concentration is presented in **Figure S1**.

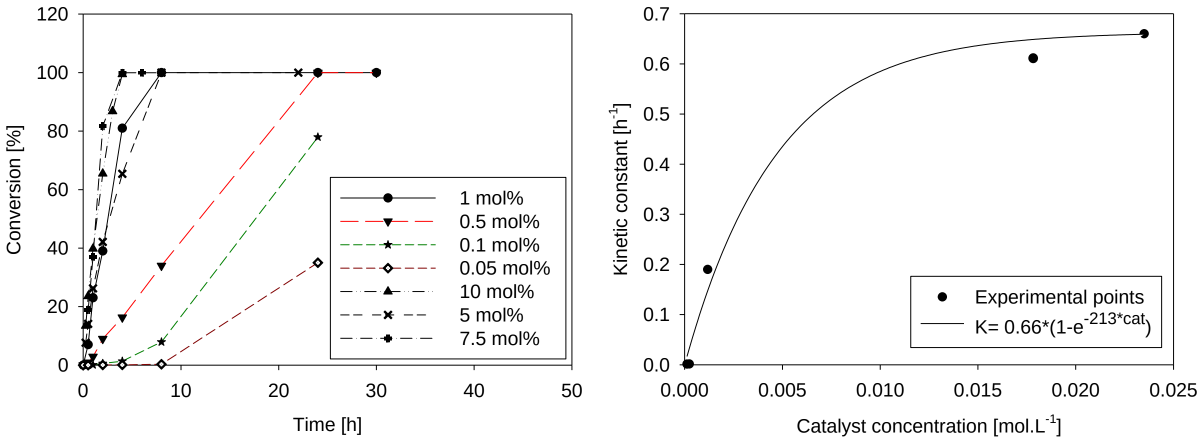

Figure S1 Conversion over time at different catalyst loadings for batch kinetic experiments of the Heck coupling reaction (left); Estimated kinetic constant (assuming first order reaction toward the limiting substrate) as a function of the catalyst concentration and the fitted exponential equation curve (right).

The rate of the Heck reaction is somewhat low compared to some reaction times in the literature within the minute range[3]. This work replicated the conditions developed by Caron et al [2] where the batch process time is 10 hours, suggesting the slow reaction confirmed by the reaction kinetics in Figure S1. Generally bromobenzenes have relatively low reactivity and require higher temperatures and longer residence times than commonly reported alternatives such as iodobenzenes. For example a study on Heck coupling of aryl halides to alkenes under segmented flow conditions has shown that the best conversion achieved at 40 min residence time and using 10 mol% catalyst at 130^{0}C was 65% for p-bromonitrobenzene, while for most bromobenzenes the conversion was within the range of 20-30% under the same conditions[4]. The conversion achieved at 90^{0}C in our continuous reactor is >95% which required longer residence times.

Continuous Heck coupling reaction combined with OSN membrane separation

Reactor set-up – PFR-m-CSTR

The set-up consisted of two reactors in series. A constant flow of the feed solution was first passed through a U-shaped PFR, placed in a heating chamber maintained at 90^{0}C. The PFR was made of 316 SS ½'' tube with a length of 0.64 m (total volume of 60 mL). The outlet of the PFR was directly connected to the inlet of the m-CSTR.

The m-CSTR[5] was made of 316 SS, could operate under high pressure (69 bar), and hold circular flat sheet membranes with an effective area of 51 cm^2. The m-CSTR was operated in a bottom-to-top permeation mode and contained a magnetic stirrer in the feed/retentate chamber. This is to ensure that any dissolved gas released from a feed stream which enters the cell will move to the top of the cell and exit through the membrane. The liquid capacity of the m-CSTR was 100 mL. Six ports surround the bottom section of the cell, and were used as inlet ports (feed) or outlet ports (permeate), or were connected to a thermocouple or a pressure gauge for temperature control and pressure monitoring . The pressure in the reactor varied with the flow rate but remained within the range of 5-15 bar. The permeate/product stream was clear and transparent since the bulk of the Pd catalyst was retained in the reactor (**Figure S2**)

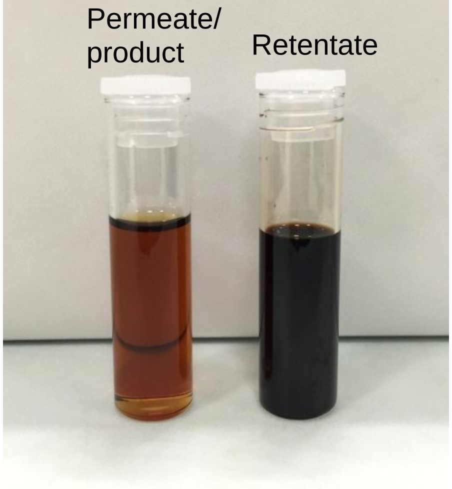

Figure S2 Image of permeate (product) and retentate from the m-CSTR

Operating procedure

For 300 mL of the initial feed solution, 7.2 g 1-Bromo-4-chloro-2-nitrobenzene (1) (0.1 mol.L^{-1}), 0.067 g $Pd(OAc)_2$ (1.0×10^{-3} mol.L^{-1}, ~ 1.0 mol %) and 0.16 g PPh_3 (2.0×10^{-3} mol.L^{-1}, ~2.0 mol %) were added into a 500 mL two-neck round bottom flask. The flask was vacuum degassed and then placed under an N_2 atmosphere. After that, and always under an N_2 atmosphere, ~ 262 mL of anhydrous DMF were added and the solution was mixed using a magnetic stirrer. Then, 32 mL of ethyl acrylate (10 equivalents, 1 mol.L^{-1}) and 5.9 mL of triethylamine (1.4 equivalents, 0.14 mol.L^{-1}) were added to the flask and mixed. The flask was then connected to the system as a feed solution and kept under an N_2 blanket (~ 0.5 bar overpressure). More feed solution was prepared throughout the running of the system by using the procedure described above but with 10 times lower catalyst and ligand concentrations. Catalyst loadings were varied throughout the continuous run in order to increase productivity and decrease residence time.

Note: The system was run initially in a batch mode. 160 mL starting solution (0.1 mol.L^{-1} 1-Bromo-4-chloro-2-nitrobenzene (1); 0.5×10^{-3} mol.L^{-1} $Pd(OAc)_2$) and corresponding amounts of the other reagents, were pumped into the PFR-m-CSTR chamber and stirred for ~ 12 hours at 90 °C (overnight). On the following day the system was started in continuous mode using a feed stream containing again 0.1 mol.L^{-1} 1-Bromo-4-chloro-2-nitrobenzene (1) and 0.5 mol % Pd (0.5 $\times10^{-3}$ mol.L^{-1} $Pd(OAc)_2$).

Membrane reactor stability study

In order to evaluate the membrane performance and stability over a prolonged period of operation whilst minimising the amount of catalyst used a 1100 hour long run was performed using catalyst loadings in the range 0.05-1mol%. The membrane performance remained reasonably stable throughout the run with catalyst rejection around 90%. The large fluctuations in the experimental results are due to instability of the HPLC pump flow. Due to the swelling of plastic parts of the HPLC check valves the pre-set flow rate was not accurate and needed readjustment which in turn

led occasionally to high flow rates overnight, when constant monitoring was not in place. Eventually the check-valves were replaced with entirely metallic ones, which substantially stabilised system performance. Overall the compatibility of the system material with the aggressive reaction feed (particularly the ethyl acrylate which caused considerable swelling of all plastic parts except the PEEK membrane) proved to be of major importance and required careful consideration.

Product isolation

Small scale batches of the Heck reaction product were also prepared. To a solution of 1-Bromo-4-chloro-2-nitrobenzene (1) (8.4 g, 35.5 mmol), $Pd(OAc)_2$ (80 mg, 0.356 mmol, 1 mol%) and PPh_3 (185 mg, 0.705 mmol, 2 mol%) in dry DMF (100 mL) was added Et_3N (6.8 mL, 48.8 mmol) and ethyl acrylate (38 mL, 356 mmol). The solution was heated at 90 °C for 23 h. Following the procedure from Caron[2], the post-reaction mixture (100 mL) was allowed to cool down and diluted with toluene (100 mL). The solution was washed with 1M HCl (100 mL) and water (2 x 100 mL). The organic phase was dried over Na_2SO_4 and the solvent removed *in vacuo* to yield a brown oil. The residue was triturated with hexane to provide the title compound. The mother liquor was concentrated, and the residue again triturated with hexane to provide further portions of the title compound. Total yield was ~72%. Isolated product was analysed by ^{1}H NMR spectroscopy. ^{1}H NMR (400 MHz, $CDCl_3$) δ 8.07 (dd, *J* = 8.9, 6.9 Hz, 2H), 7.70 – 7.54 (2H), 6.38 (d, *J* = 15.8 Hz, 1H), 4.32 (q, *J* = 7.1 Hz, 2H), 1.37 (t, *J* = 7.1 Hz, 3H). Data are consistent with that reported previously[2]. The isolated product was used for preparing artificial solutions for the kinetic studies and the preliminary continuous experiments on the second reaction.

The crude product from the continuous reaction was also analysed by ^{1}H NMR spectroscopy, and was found to contain <20% impurities, consisting mainly of the starting 1-Bromo-4-chloro-2-nitrobenzene (1) (excluding the triethylamine hydrobromide) (**Figure S3**).

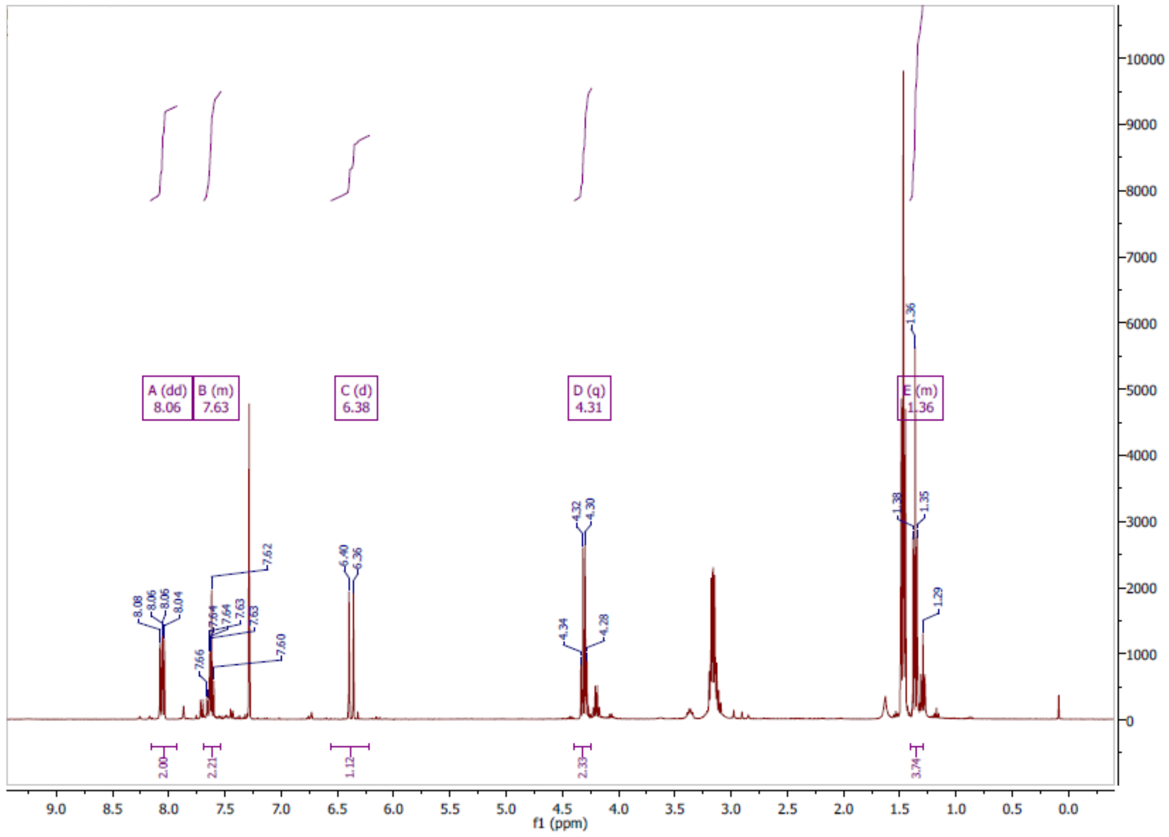

Figure S3 ^{1}H NMR spectra of the isolated crude product from the continuous Heck reaction.

Reduction Reaction - Product 2 - Ethyl (*E*)-3-(2-amino-4-chlorophenyl)acrylate (3)[2]

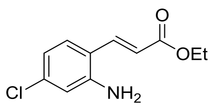

Reduction reaction kinetic studies

The kinetic studies were performed as series of batch experiments on a reaction carousel (Radleys, UK). Reactions at each ethanol: DMF composition were run in duplicates. The product of the Heck reaction, the Ethyl (*E*)-3-(4-chloro-2-nitrophenyl)acrylate (2) (0.214 g, 0.838 mmol) is dissolved in ethanol, or the corresponding ethanol:DMF mixture (3 mL) and water (0.85 mL) and the carousel tube is heated to 85°C, then iron powder (325 mesh) (0.141 g, 2.52 mmol) and ammonium chloride (0.027 g, 0.50 mmol) were added.

The reaction is very fast and small samples 0.1 mL were taken frequently at about 2-5 min intervals to determine the reaction kinetic. Samples were diluted with 0.4 mL ethanol and analysed by GC. Typically the reaction got to completion within ~1 hour.

Continuous reduction reaction

A stainless steel column (240 x10 mm) was filled with 69 g iron powder (325 mesh, density 7.86 $g.mL^{-1}$), void fraction ~0.47. The column was placed into a heating chamber, maintained at 85°C. The product stream from the membrane cascade was mixed with 0.59M NH_4Cl aqueous solution in ratio 3.5:1 and pumped into the column via an HPLC pump. The pump flow rate was varied to match the flow rate from the cascade.

Product isolation

The postreaction mixture was filtered through Celite©, and the ethanol removed *in vacuo*. The residue was diluted with toluene, extracted with water, dried over Na_2SO_4, and the solvent removed *in vacuo*. The resulting oil was purified by flash column chromatography (hexane:EtOAc 9:1) to provide the title compound. R_f = 0.2 (hexane:EtOAc 9:1) ^{1}H NMR (400 MHz, Chloroform-d) δ 7.75 (d, *J* = 15.8 Hz, 1H), 7.32 (d, *J* = 8.3 Hz, 1H), 6.78 – 6.71 (2H), 6.35 (d, *J* = 15.8 Hz, 1H), 4.29 (q, *J* = 7.2 Hz, 2H), 4.07 (s, 2H), 1.36 (t, *J* = 7.2 Hz, 4H). Data are consistent with that reported previously[2].

The crude product from the continuous reaction was also analysed by ^{1}H NMR spectroscopy, and was found to contain <20% impurities (excluding the triethylamine hydrobromide, that was carried trough from the first reaction), consisting of the reduced 1-Bromo-4-chloro-2-nitrobenzene (1) (2-Bromo-5-chloroaniline) – (**FigureS4**)

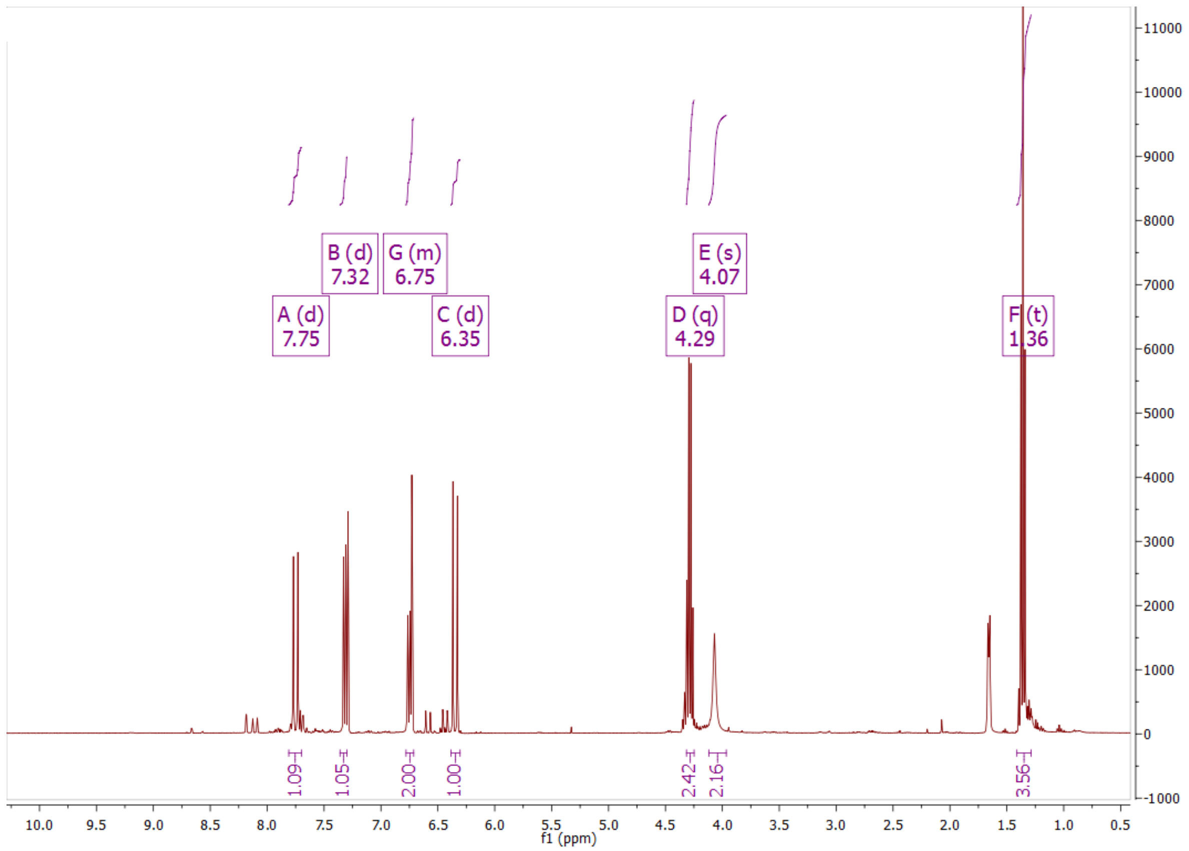

Figure S4 ^{1}H NMR spectra of the isolated crude product from the continuous reduction reaction utilising the membrane cascade stream as an inlet substrate stream.

Analysis

Conversion analysis via gas chromatography

An Agilent 6890 Series II gas chromatograph equipped with a HP - 5 column (5 % phenyl methyl siloxane; capillary: 30m×0.530 mm×1.50 µm) and a flame ionization detector (FID) was used for determining the conversion of limiting substrate to product in both reactions. A separate calibration curve was prepared for each product and each substrate and the conversion calculated as Conversion = Concentration product/(Concentration product + Concentration substrate). The programme ran from 40 °C (1 min hold) to 200 °C with a ramp of 15 °C.min^{-1}.

Palladium analysis

0.5 mL feed, permeate and retentate samples were heated at 90 °C on a hotplate stirrer. After complete drying, 1.5 mL of aqua regia (nitric acid and hydrochloric acid 1:3 v/v) was added to each dried sample to digest the organic content (digestion within ~ 24 hours). Each sample was then diluted in 10 mL centrifuge tubes with distilled water and mixed (the small residual organic matter was found not to interfere with the analysis). The samples were analysed using *Inductively Coupled Plasma* Optical Emission Spectrometry (ICP-OES) on a Perkin-Elmer Optima 2000DV spectrometer and compared against a calibration curve of 2 ppm, 5 ppm and 10 ppm palladium standard samples.

Membrane Cascade

Membrane selection procedure

To select the most suitable membrane for the cascade preliminary membrane screening was performed using a small test rig, consisting of 2 circular membrane cells, holding 14 cm^2 membrane disk an HPLC pump and a 0.1 L reservoir (**FigureS5**). Artificial solution of 10 $g.L^{-1}$ (0.03 $mol.L^{-1}$) Ethyl (*E*)-3-(4-chloro-2-nitrophenyl)acrylate (2) - product of the Heck reaction, and 6 $g.L^{-1}$ (0.03 $mol.L^{-1}$) Triethylamine hydrobromide - salt dissolved in solvent mixture of 20% DMF and 80% ethanol was recirculated through the cells under 30 bar pressure maintained via relief valve/back pressure regulator. Feed and permeate samples were collected over time and analysed. Since the product is small MW (255.5 Da) only very tight membranes were screened (both commercially available and prepared at Imperial College). A summary of the membranes screened with their permeance and rejection is presented in **Table S1**

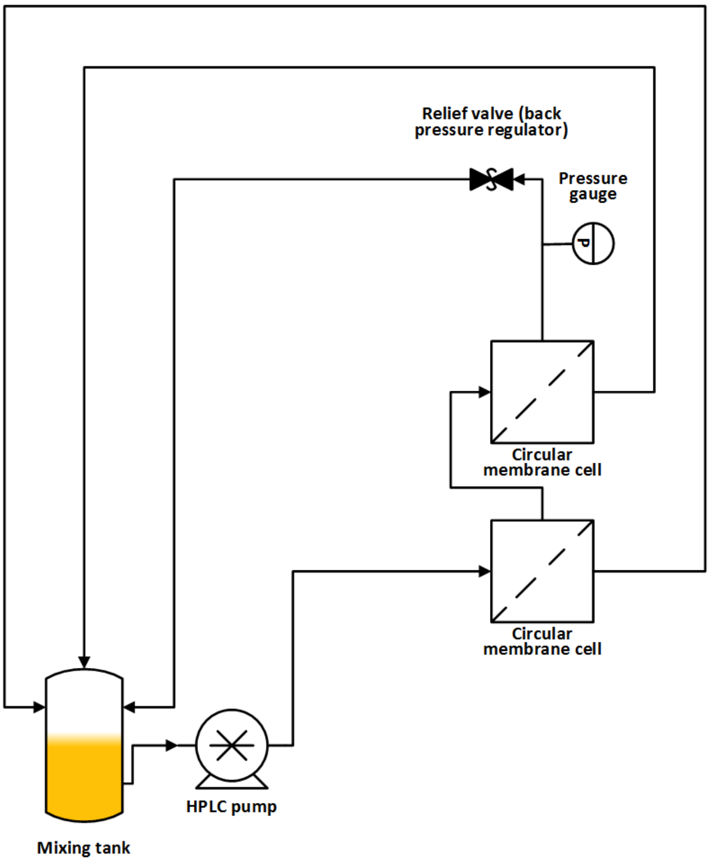

FigureS5 Schematic representation of the membrane screening rig.

Table S1 Permeance and rejection of membranes screened for possible operation in the membrane cascade**.**

Membrane	Specification	Product rejection [%]	Salt rejection [%]	Permeance $L.m^{-2}h^{-1}bar^{-1}$
24% PBI	24% polybenzimidazole[6]	44	71	0.42
TFC1	Thin film composite membrane- PA prepared on cross-linked PI using Tris(2-aminoethyl)amine/ Trimesoyl chloride[7]	84	88	0.04
TFC2	Thin film composite membrane- PA prepared on cross-linked PI using Trimesoyl chloride /*m*-phenylenediamine[7]	83	-	0.20
Duramem 150	Commercial membrane from Evonik Industries	96	98	0.05

Duramem 150, although exhibiting low permeance, showed the best rejection for the product and was chosen for further study in the membrane cascade.

Experimental setup – membrane cascade

Schematic representation of the membrane cascade is shown in **Figure 1**, and a more detailed drawing of a single stage (stage 1) is presented in **FigureS6** while further information about flow rates and stream compositions is provided in **FigureS7**. The cascade consists of 3 stages connected in series. Each stage (hold-up volume 0.25L) consists of a 0.1 L mixing tank (A) where the Heck post reaction mixture (stage 1) or replacing solvent (stage 3) were constantly fed in. An HPLC pump (B) supplies feed from the tank into the corresponding stage of the cascade. The cascade operated in a counter current mode. Permeate from each stage is measured and controlled via a coriolis mass flow controller (F) and is fed into the mixing tank of the previous stage. Permeate from the first stage is fed directly into a recovery stage (D) in order to increase the product yield[8]. Each stage (except the recovery stage) comprises 3 circular cross-flow cells (C) (~0.04L holdup volume each) connected in series. Each circular cell holds a membrane disk with an area of 51 cm^2. Mixing is provided by vigorously recirculating the retentate of each stage via a gear pump (80 $L.h^{-1}$, residence time per cell of ~2 s). The pressure at each stage is controlled independently via a back pressure regulator (E). An overflow stream from the mixing tank of each stage is transferred by gravity into the mixing tank of the next stage. The product stream in the exchange solvent is collected as overflow stream from the mixing tank of stage 3. The cascade operating parameters, pressures and flow rates were preliminary estimated using a dynamic model based on the mass balance equations, permeances and rejections determined by independent experiments. This simple configuration allows for relatively easy control of the cascade operation. Depending on the membrane permeance the number of cells per stage can be easily varied in order to obtain desired permeate flows. A single membrane cell, similar to the m-CSTR reactor described in the continuous Heck reaction set-up section was used for the recovery stage.

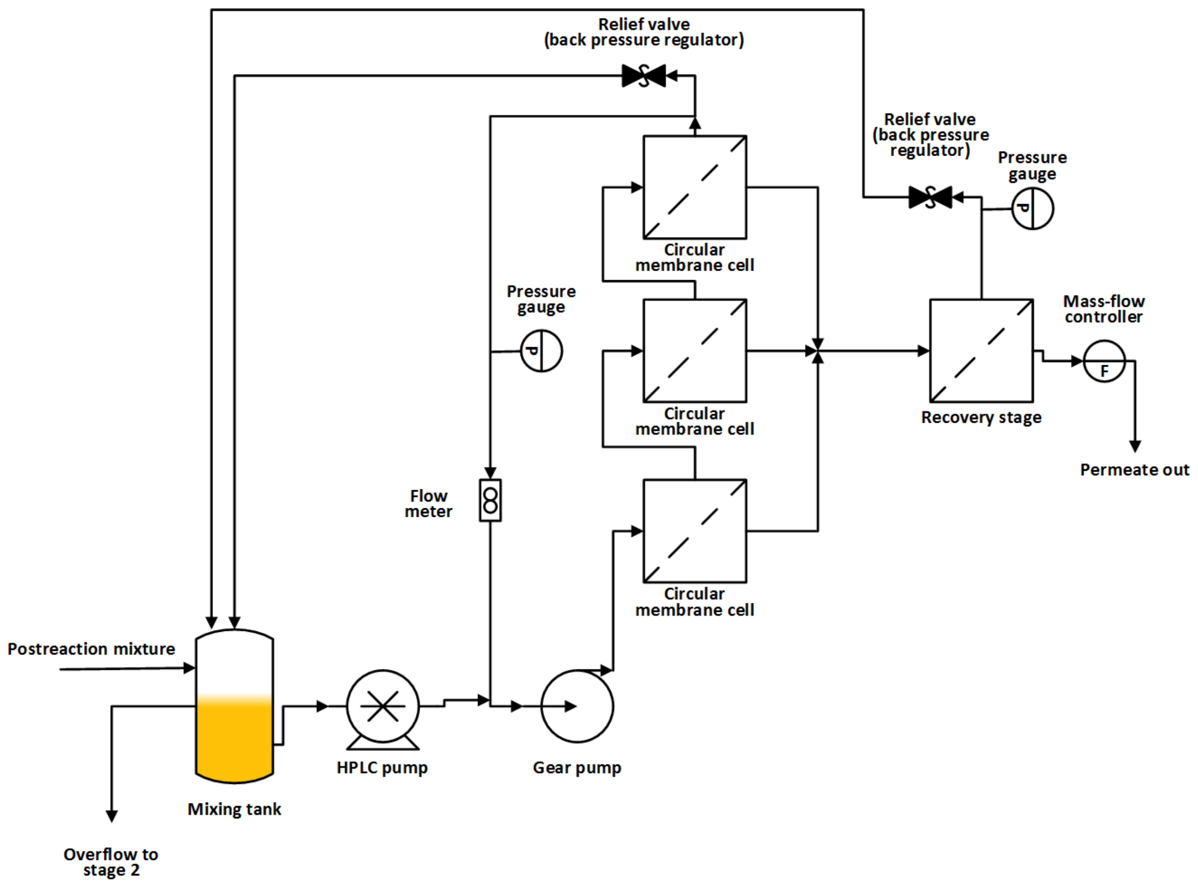

FigureS6 Schematic representation of Stage1 of the membrane cascade.

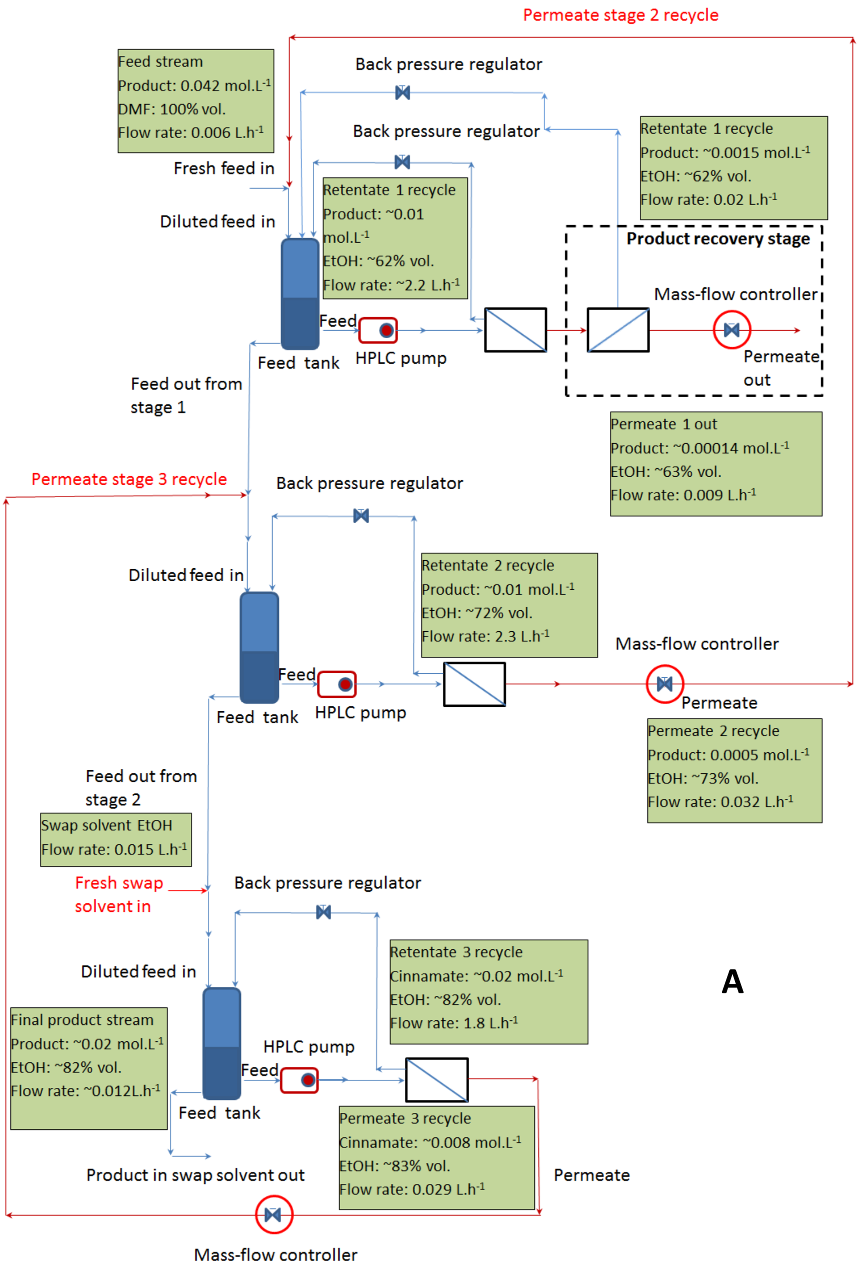
Permeate stage 2 recycle
Feed stream
Product: 0.042 mol.L-1
DMF: 100% vol.
Flow rate: 0.006 L.h-1
Back pressure regulator
Back pressure regulator
Fresh feed in
Diluted feed in
Retentate 1 recycle
Product: ~0.01 mol.L-1
EtOH: ~62% vol.
Flow rate: ~2.2 L.h-1
Retentate 1 recycle
Product: ~0.0015 mol.L-1
EtOH: ~62% vol.
Flow rate: 0.02 L.h-1
Product recovery stage
Mass-flow controller
Feed
Feed tank
HPLC pump
Permeate out
Feed out from stage 1
Permeate 1 out
Product: ~0.00014 mol.L-1
EtOH: ~63% vol.
Flow rate: 0.009 L.h-1
Permeate stage 3 recycle
Back pressure regulator
Diluted feed in
Retentate 2 recycle
Product: ~0.01 mol.L-1
EtOH: ~72% vol.
Flow rate: 2.3 L.h-1
Mass-flow controller
Feed
Feed tank
HPLC pump
Permeate
Permeate 2 recycle
Product: 0.0005 mol.L-1
EtOH: ~73% vol.
Flow rate: 0.032 L.h-1
Feed out from stage 2
Swap solvent EtOH
Flow rate: 0.015 L.h-1
Fresh swap solvent in
Back pressure regulator
Diluted feed in
Retentate 3 recycle
Cinnamate: ~0.02 mol.L-1
EtOH: ~82% vol.
Flow rate: 1.8 L.h-1
A
Final product stream
Product: ~0.02 mol.L-1
EtOH: ~82% vol.
Flow rate: ~0.012L.h-1
HPLC pump
Feed
Feed tank
Permeate 3 recycle
Cinnamate: ~0.008 mol.L-1
EtOH: ~83% vol.
Flow rate: 0.029 L.h-1
Product in swap solvent out
Permeate
Mass-flow controller

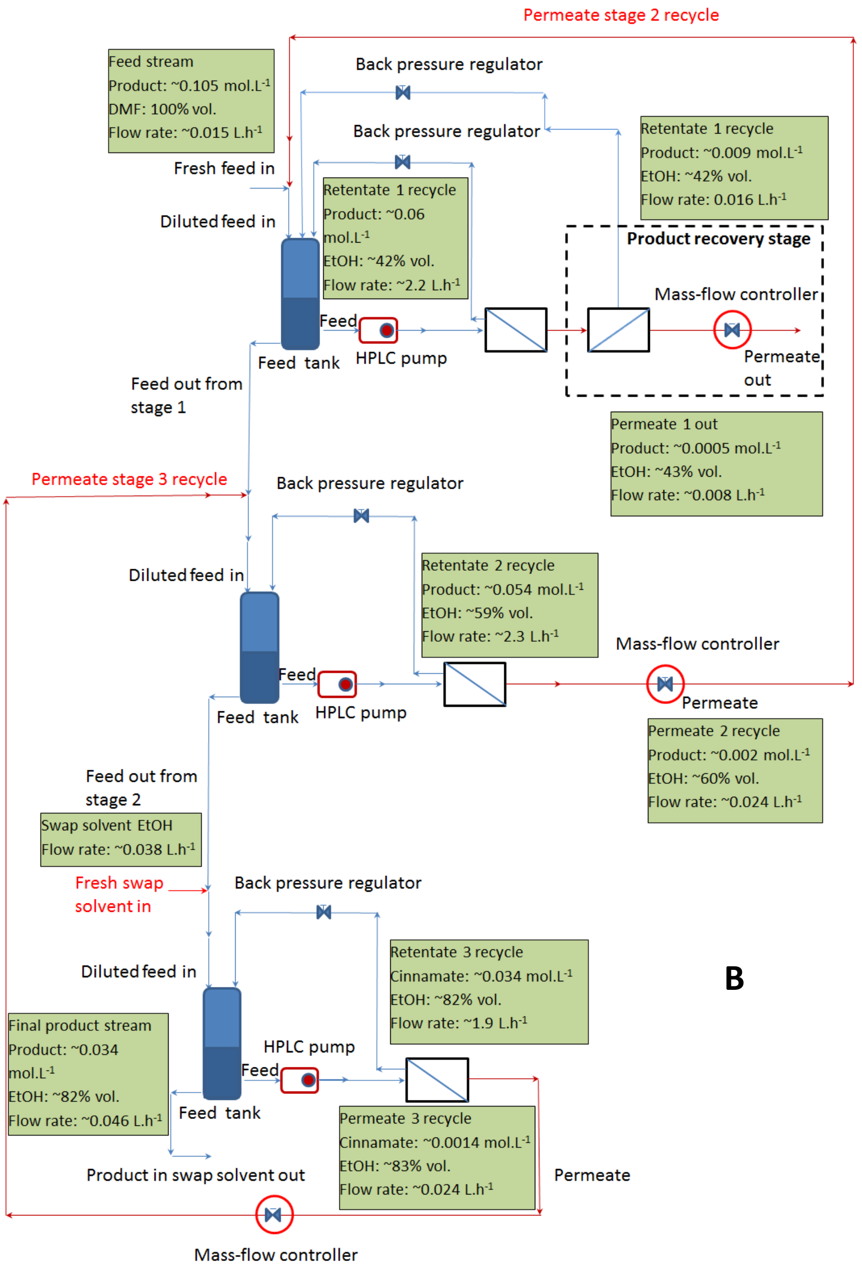

FigureS7 Schematic representation of the stream flow rates and compositions used in the membrane cascade solvent exchange continuous runs. A - run utilising postreaction stream obtained at 53h residence time in the PFR-m-CSTR; B - run utilising postreaction stream obtained at 10h residence time in the PFR-m-CSTR.

Options for improving continuous process performance

As described in the main body of the manuscript the membrane cascade reached steady state within ~100 hours (when started at 0 concentration of the product in all

stages) and the final product stream was diluted 2-3 times as compared to the feed stream. We wanted to evaluate if these undesired phenomena could be avoided.

Undoubtedly the hold-up volume of the cascade plays an important role for the response time. The model estimations suggest that if we are able to reduce the volume of each stage of the current cascade (except the recovery stage) from 0.25 to 0.1 L the response time will decrease from ~100h to ~45h without sacrificing the solvent exchange or the product recovery (**Figure S8**). Alternatively the product dilution could be reduced by using a membrane with the same rejection but higher permeance. For example a membrane with a permeance 4 times higher than the current membrane would completely eliminate product dilution and even improve the solvent exchange ratio retaining the same product recovery (**Figure S9**). Optimisation studies on the membrane cascade are beyond the scope of the current work, but these estimates indicate that the cascade performance can be further improved.

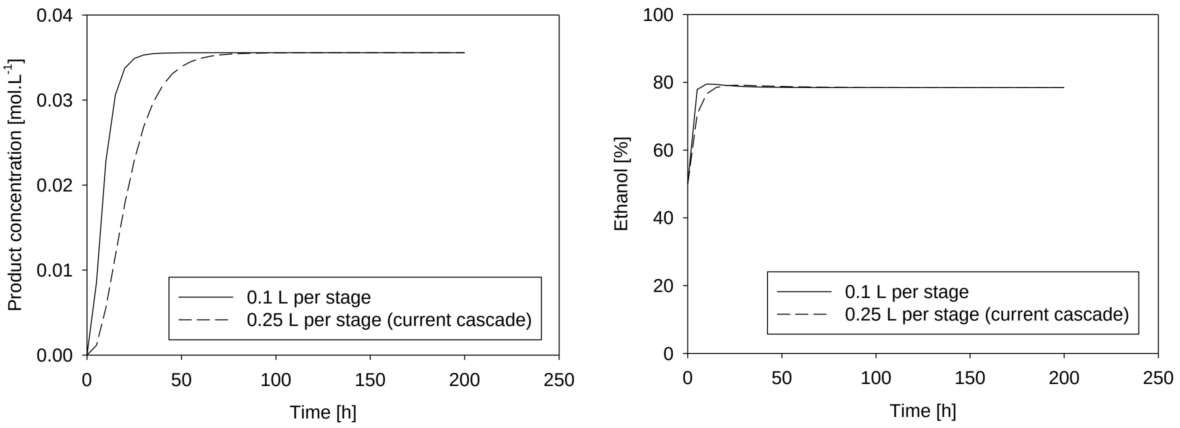

Figure S8 Effect of the stage volume on the product (left) and ethanol (right) concentration in the final product stream over time. Simulations are performed for 3 stage cascade with product recovery stage utilising 0.1 mol.L^{-1} feed in at 0.015 L.h^{-1} and swap solvent in at 0.0375 L.h^{-1}. Estimated product recovery at steady state is ~99.8%.

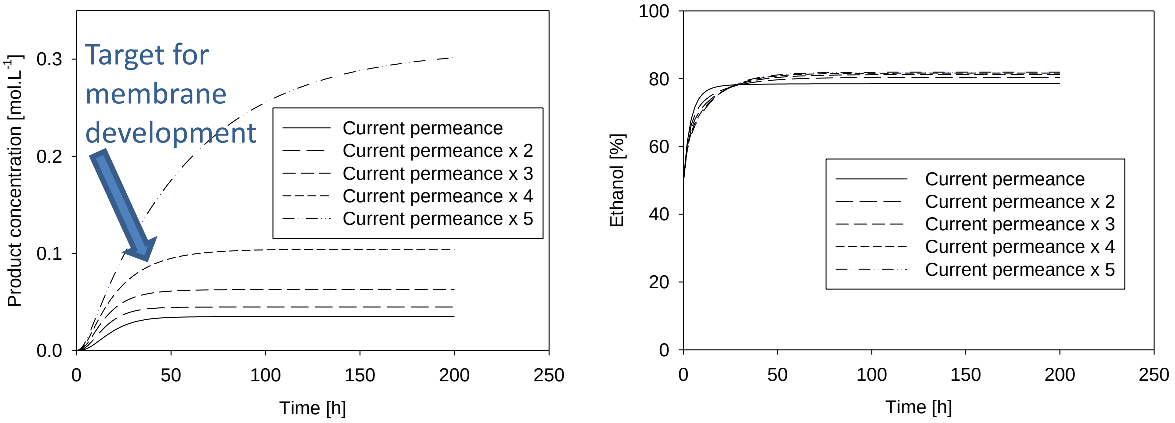

Figure S9 Effect of membrane permeance on the product (left) and ethanol (right) concentration in the final product stream over time. Simulations are performed for 3 stage cascade with product recovery stage utilising 0.1 mol.L^{-1} feed in at 0.015 L.h^{-1} and swap solvent in at 0.0375 L.h^{-1}. Estimated product recovery at steady state is ~99.8%.

Apart from improving the membrane cascade performance, other options for overall process improvement could be made. For example an adsorption column placed after the continuous Heck reaction membrane unit would improve the purity of the product. Alternatively an additional continuous membrane purification unit can be placed before or after the solvent exchange membrane cascade. As can be seen from **Table S1** the side product triethylamine hydrobromide is also well retained by the membranes. Interestingly the salt is much better retained by the 24% PBI membrane than the main product (71% vs. 44% rejection). This is an advantage and could be used to design a continuous membrane purification unit similar to the one reported earlier[9] which is able to retain the impurity and permeates the desired product through. Finally the product from the reduction reaction can be crystallised in a continuous MSMPR crystallization unit with integrated nanofiltration membrane recycle for enhanced yield and purity[10]. Conceptual design of a multiple reaction continuous process with different membrane units embedded is presented in **Figure S10**.

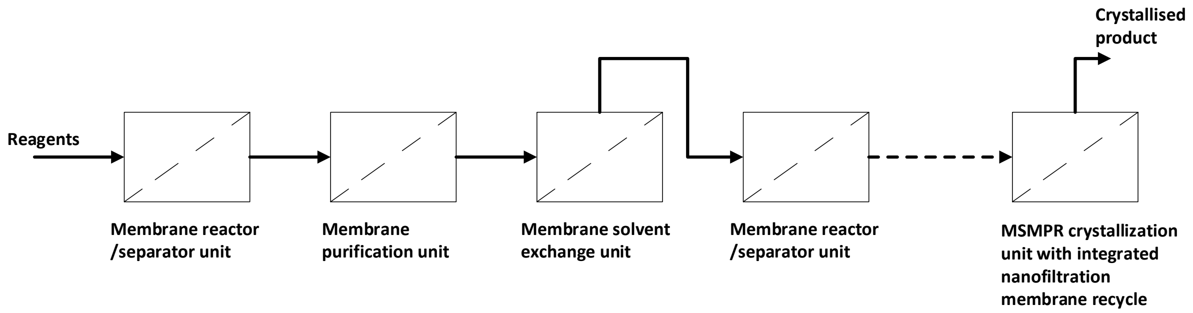

Figure S10 Conceptual design of a multiple reaction continuous process with different membrane units embedded.

Mathematical Model

Membrane cascade

To evaluate and predict cascade performance a simple model based on mass balance equations was developed (Eqs. 1-29). The model is based on the following assumptions i) the concentrations in the mixing tank and the corresponding cascade stage are the same – this assumption was validated experimentally; ii) the membrane has no separation properties toward the solvents ethanol and DMF, i.e. the ethanol and DMF concentrations in the retentate and permeate are the same; although small separation (less than 1% difference) seemed to occur for simplicity it was not taken into account; iii) it was difficult to quantify the osmotic pressure with so many compounds present in the postreaction mixture, instead the apparent permeance and rejection determined during the membrane selection experiments (**Table S1**) was used for preliminary cascade optimisation and selection of operating pressures. The optimisation procedure was performed using gPROMs dynamic simulator (gOPT) with a target function for minimum solvent volumes usage (Fsolvent/Fin = minimum). Pressures and feed to swap solvent ratios determined were further used in the cascade experimental runs. For the final calculations presented in **Figure 4** the apparent permeance at each stage was determined experimentally during the run.

EQUATIONS

Stage 1

$$V_1 \times \frac{dC_{Pr_1}}{dt} = F_{in} \times C_{Pr_{in}} + R_{Rec} \times C_{Pr_{Rec}} + P_2 \times C_{Pr_{P_2}} - F_1 \times C_{Pr_1} - P_1 \times C_{Pr_{P_1}} \quad 1$$

$$V_1 \times \frac{dC_{EtOH_1}}{dt} = F_{in} \times C_{EtOH_{in}} + R_{Rec} \times C_{EtOH_{Rec}} + P_2 \times C_{EtOH_{P_2}} - F_1 \times C_{EtOH_1} - P_1 \times C_{EtOH_{P_1}} \quad 2$$

$$F_{in} + R_{Rec} + P_2 - F_1 - P_1 = 0 \quad 3$$

$$C_{Pr_{P_1}} = C_{Pr_1} \times \left(1 - Rej_{Pr_1}\right) \quad 4$$

$$C_{EtOH_{P_1}} + C_{DMF_{P_1}} = 1 \quad 5$$

$$C_{EtOH_1} + C_{DMF_1} = 1 \quad 6$$

$$P_1 = Perm_1 \times Area_1 \times \Delta p_1 \quad 7$$

Stage 2

$$V_2 \times \frac{dC_{Pr_2}}{dt} = F_1 \times C_{Pr_1} + P_3 \times C_{Pr_{P_3}} - F_2 \times C_{Pr_2} - P_2 \times C_{Pr_{P_2}} \quad 8$$

$$V_2 \times \frac{dC_{EtOH_2}}{dt} = F_1 \times C_{EtOH_1} + P_3 \times C_{EtOH_{P_3}} - F_2 \times C_{EtOH_2} - P_2 \times C_{EtOH_{P_2}} \quad 9$$

$$F_1 + P_3 - F_2 - P_2 = 0 \quad 10$$

$$C_{Pr_{P_2}} = C_{Pr_2} \times \left(1 - Rej_{Pr_2}\right) \quad 11$$

$$C_{EtOH_{P_2}} + C_{DMF_{P_2}} = 1 \quad 12$$

$$C_{EtOH_2} + C_{DMF_2} = 1 \quad 13$$

$$P_2 = Perm_2 \times Area_2 \times \Delta p_2 \quad 14$$

Stage 3

$$V_3 \times \frac{dC_{Pr_3}}{dt} = F_2 \times C_{Pr_2} + F_{EtOH} \times C_{Pr_{EtOH}} - F_3 \times C_{Pr_3} - P_3 \times C_{Pr_{P_3}} \quad 15$$

$$V_3 \times \frac{dC_{EtOH_3}}{dt} = F_2 \times C_{EtOH_2} + F_{EtOH} \times C_{EtOH_{EtOH}} - F_3 \times C_{EtOH_3} - P_3 \times C_{EtOH_{P_3}} \quad 16$$

$$F_2 + F_{EtOH} - F_3 - P_3 = 0 \quad 17$$

$$C_{Pr_{P_3}} = C_{Pr_3} \times \left(1 - Rej_{Pr_3}\right) \quad 18$$

$$C_{EtOH_{P_3}} + C_{DMF_{P_3}} = 1 \quad 19$$

$$C_{EtOH_3} + C_{DMF_3} = 1 \quad 20$$

$$P_3 = Perm_3 \times Area_3 \times \Delta p_3 \quad 21$$

Recovery stage

$$V_{Rec} \times \frac{dC_{Pr_{Rec}}}{dt} = P_1 \times C_{Pr_{P_1}} - R_{Rec} \times C_{Pr_{Rec}} - P_{Rec} \times C_{Pr_{P_{Rec}}} \quad 22$$

$$V_{Rec} \times \frac{dC_{EtOH_{Rec}}}{dt} = P_1 \times C_{EtOH_{P_1}} - R_{Rec} \times C_{EtOH_{Rec}} - P_{Rec} \times C_{EtOH_{P_{Rec}}} \quad 23$$

$$P_1 - R_{Rec} - P_{Rec3}=0 \quad 24$$

$$C_{Pr_{P_{Rec}}} = C_{Pr_{Rec}} \times \left(1 - Rej_{Pr_{Rec}}\right) \quad 25$$

$$C_{EtOH_{P_{Rec}}} + C_{DMF_{P_{Rec}}} = 1 \quad 26$$

$$C_{EtOH_{Rec}} + C_{DMF_{Rec}} = 1 \quad 27$$

$$P_{Rec} = Perm_{Rec} \times Area_{Rec} \times \Delta p_{Rec} \quad 28$$

$$Recovery = F_3 \times C_{Pr_3}/F_{in} \times C_{Pr_{in}} \quad 29$$

Heck reaction

The model describing PFR-m-CSTR system is based on the mass-balance equations (Eqs. 30-37). The effect of catalyst concentration was accounted for via the catalyst rejection, estimated at ~90%, and an algebraic equation describing the kinetic constant as a function of catalyst concentration. For simplicity the reaction kinetic was approximated to a first order reaction toward the limiting substrate 1-Bromo-4-chloro-2-nitrobenzene and the kinetic constant at each catalyst loading was determined using the isolation method. An exponential curve fitting was applied in order to describe kinetic constant as a function of the catalyst concentration (see Kinetic study section SI). This is a simplified approach for the complexity of Pd catalysed reaction, however it describes satisfactorily the reactor performance for the scopes of this work. The rejections were determined from an independent experimental filtration run at 90^0C and 30 bar pressure in the m-CSTR equipped with a PEEK membrane. An artificial solution of substrate -1-Bromo-4-chloro-2-nitrobenzene (1), product - Ethyl (*E*)-3-(4-chloro-2-nitrophenyl)acrylate (2) and salt - Triethylamine hydrobromide 0.01 mol.L^{-1} each in DMF was used and the rejections were determined as following: 9% substrate, 24% product and 54% salt. This experiment together with the kinetic studies was carried out in order to be able to make a reasonable prediction of the PFR-m-CSTR reactor performance. Most importantly our previous studies[11] have shown that side product (salt) solubility

could be a major issue during the continuous experiment causing salt accumulation and consequent precipitation, reactor clogging and over pressurising. The salt solubility at 90^{0}C in DMF was also determined at ~0.56 mol.L^{-1}. Using equation 38 two substrate concentrations (0.1 and 0.2 mol.L^{-1}) were theoretically evaluated to verify whether the salt concentration in the m-CSTR reactor will remain below the solubility limit during continuous run. To simulate the extreme case it was assumed instantaneous 100% conversion of substrate to product and salt. As can be seen from Figure S11 for both substrate concentrations the salt in the reactor remains below its solubility limit and it was safe to perform the experiment.

EQUATIONS

$$Conv_{PFR} = 1 - e^{\left(-\left(0.6635\times\left(1-e^{-213\times C_{CAT_{in}}}\right)\right)\times\left({V_{PFR}}/{F}\right)\right)} \qquad 30$$

$$V_{CSTR} \times \frac{dC_{CAT}}{dt} = F \times C_{CAT_{in}} - F \times C_{CAT} \times (1 - Rej_{CAT}) \qquad 31$$

$$V_{CSTR} \times \frac{dC_S}{dt} = F \times C_{S_{in}} \times (1 - Conv_{PFR}) - V_{CSTR} \times 0.6635 \times \left(1 - e^{-213\times C_{CAT_{in}}}\right) \times C_S - F \times C_S \times (1 - Rej_S) \qquad 32$$

$$V_{CSTR} \times \frac{dC_{Pr}}{dt} = F \times C_{S_{in}} \times Conv_{PFR} + V_{CSTR} \times 0.6635 \times \left(1 - e^{-213\times C_{CAT_{in}}}\right) \times C_S - F \times C_{Pr} \times (1 - Rej_{Pr}) \qquad 33$$

$$C_{CAT_{out}} = C_{CAT} \times (1 - Rej_{CAT}) \qquad 34$$

$$C_{S_{out}} = C_S \times (1 - Rej_S) \qquad 35$$

$$C_{Pr_{out}} = C_{Pr} \times (1 - Rej_{Pr}) \qquad 36$$

$$Conv = \frac{C_{Pr_{out}}}{C_{Pr_{out}} + C_{S_{out}}} \qquad 37$$

$$V_{CSTR} \times \frac{dC_{SALT}}{dt} = F \times C_{S_{in}} - F \times C_{SALT} \times (1 - Rej_{SALT}) \qquad 38$$

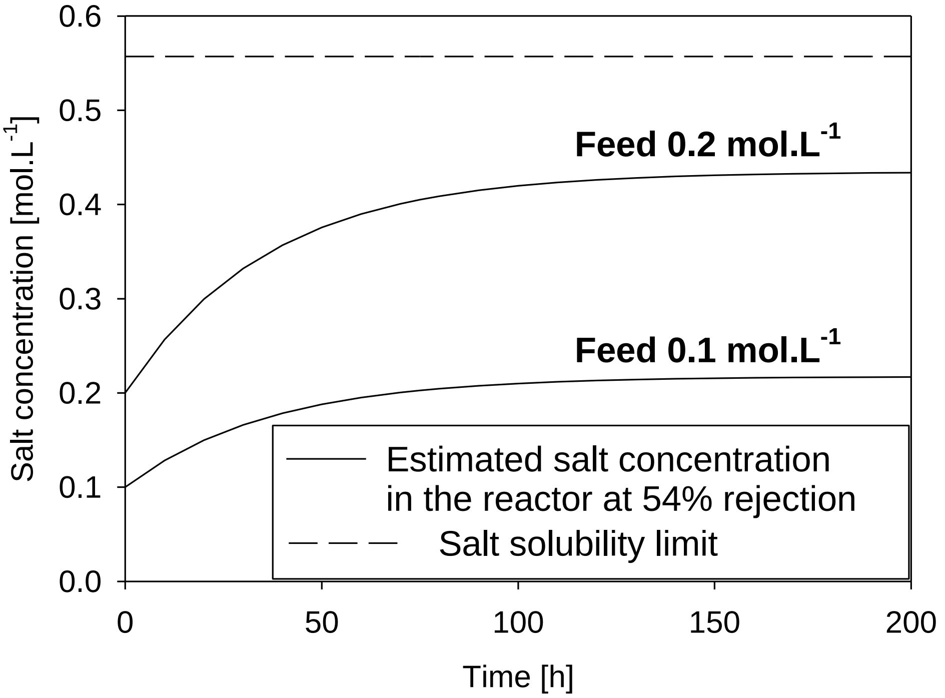

Figure S11 Salt concentration with time at two different 1-Bromo-4-chloro-2-nitrobenzene (1) concentrations in the feed solution – 0.1 mol.L^{-1} and 0.2 mol.L^{-1}. The estimation for both concentrations was performed using a flow rate of 0.1 mL.min^{-1} and a rejection of the salt of 54 %. Note that the salt concentration at time 0 does not start from null because the reactor is prefilled with reaction solution and kept at 90^{0}C until full conversion in batch before starting the continuous run.

Nomenclature

Area – membrane area, m^2

$C_{i,j}$ – concentration of compound i in a stream j, e.g. $c_{Pr_{P_3}}$ concentration of product in permeate from stage 3, $g.L^{-1}$ or $L.L^{-1}$ for solvents.

F_{in} – feed stream entering the cascade, $L.h^{-1}$

F_i – feed entering a corresponding stage of the cascade from previous stage i, $L.h^{-1}$

$Perm_i$ – membrane permeance of stage i, $L.m^{-2}.h^{-1}.bar^{-1}$

P_i – permeate from stage i, $L.h^{-1}$

R_i – retentate from stage i, $L.h^{-1}$

Rej_i – rejection of compound i, -

t – time, h

V_i – volume of stage i, L

Δp_i – trans-membrane pressure in stage i, bar

Abbreviations

CAT – catalyst

Conv - conversion

Pr – product

S – substrate

References

[1] J. da Silva Burgal, L. G. Peeva, S. Kumbharkar, A. Livingston, *Journal of Membrane Science* **2015**, *479*, 105-116.

[2] S. Caron, E. Vazquez, R. W. Stevens, K. Nakao, H. Koike, Y. Murata, *The Journal of Organic Chemistry* **2003**, *68*, 4104-4107.

[3] T. Noel, S. L. Buchwald, *Chemical Society Reviews* **2011**, *40*, 5010-5029.

[4] B. Ahmed-Omer, D. A. Barrow, T. Wirth, *Tetrahedron Lett.* **2009**, *50*, 3352-3355.

[5] J. C.-T. Lin, A. G. Livingston, *Chemical Engineering Science* **2007**, *62*, 2728-2736.

[6] I. B. Valtcheva, S. C. Kumbharkar, J. F. Kim, Y. Bhole, A. G. Livingston, *Journal of Membrane Science* **2014**, *457*, 62-72.

[7] M. F. Jimenez Solomon, Y. Bhole, A. G. Livingston, *Journal of Membrane Science* **2012**, *423–424*, 371-382.

[8] J. F. Kim, A. M. Freitas da Silva, I. B. Valtcheva, A. G. Livingston, *Separation and Purification Technology* **2013**, *116*, 277-286.

[9] L. Peeva, J. d. S. Burgal, I. Valtcheva, A. G. Livingston, *Chemical Engineering Science* **2014**, *116*, 183-194.

[10] S. Ferguson, F. Ortner, J. Quon, L. Peeva, A. Livingston, B. L. Trout, A. S. Myerson, *Crystal Growth & Design* **2014**, *14*, 617-627.

[11] L. Peeva, J. da Silva Burgal, S. Vartak, A. G. Livingston, *Journal of Catalysis* **2013**, *306*, 190-201.
